# Prosets: a new financing instrument to deliver a durable net zero transition

**DOI:** 10.1007/s10584-022-03423-x

**Published:** 2022-09-28

**Authors:** Eli Mitchell-Larson, Myles Allen

**Affiliations:** 1 Oxford Net Zero & School of Geography and the Environment, University of Oxford, Oxford, UK; 2 Oxford Net Zero, School of Geography and the Environment & Department of Physics, University of Oxford, Oxford, UK

**Keywords:** Carbon removal, Durable net zero, Compensation claims, Carbon credit

## Abstract

Interest in carbon offsetting is resurging among companies and institutions, but the vast majority of existing offerings fail to enable a credible transition to a durable net zero emission state. A clear definition of what makes an offsetting product “net zero compliant” is needed. We introduce the “proset”, a new form of composite carbon credit in which the fraction of carbon allocated to geological-timescale storage options increases progressively, reaching 100% by the target net zero date, generating predictable demand for effectively permanent CO_2_ storage while making the most of the near-term opportunities provided by nature-based climate solutions, all at an affordable cost to the purchaser.

Is carbon offsetting a credible way of compensating for emissions from fossil fuels? While firms, investors, governments, non-state actors, and academics alike have wrestled with how to approach carbon offsetting for nearly two decades, this specific question is rarely satisfactorily answered. Even before the COVID-19 pandemic induced a temporary dip in global emissions, a new offsetting boom was underway, characterised by increasing purchase volumes (Gross et al. 2019; Laville 2019) and growing interest in nascent carbon removal methods (Izikowitz 2021; Joppa 2020). Organisations are increasingly committing to achieve net zero emissions of carbon dioxide (CO_2_) before mid-century or as early as 2030 (B Corp 2019). Consensus is building that these plans must prioritise the absolute elimination of emissions (SBTi 2021), but a subset of “hard-to-abate” emissions (Davis et al. 2018) from sectors including long-haul air travel and cement and steel production will need to be credibly compensated for with carbon credits in order to substantiate any net zero claims (rather than absolute zero claims). It will also become critical that organisation-level net zero plans align with the goal of so-called durable net zero global emissions, a state in which emissions from each carbon stock (lithosphere, biosphere) are balanced with removals that store carbon into sinks with comparable durability, like-for-like (Fankhauser et al. 2022; Worthington et al. 2021). This suggests the need for differentiated treatment of fossil fuel and biogenic emissions, as contemplated in the European Union’s climate strategy. This paper proposes an appropriate compensation mechanism for fossil fuel emissions first and foremost; the mechanism may not be appropriate, or may need to be adapted, to address biogenic emissions.

For organisations seeking to deliver durable net zero in the coming decades before having eliminated all fossil fuel emissions, their only available options apart from ceasing these activities entirely (Allwood et al. 2019) are to first reduce or substitute the activity with lower-carbon alternatives to the extent possible, and then attempt to compensate for the impact of any residual fossil emissions with the voluntary purchase of carbon credits. The bulk of offsetting options available today are either credits that avoid emissions through investment in alternatives to fossil fuels, which as the cost of renewable energy declines face increasing challenges over non-additionality (whether the benefits “would have occurred anyway”), or nature-based climate solutions, including emission reductions through avoided damage to ecosystems, or carbon removal through forestation and other nature-based sequestration (59% of carbon offset credits originating in the 2015–2020 period come from forestry and land use projects, and only ~ 3% of all such credits were removals (Mitchell-Larson and Bushman 2021)). All of the avoidance and reduction activities that underpin these carbon credits must be scaled up and financed through a variety of means beyond carbon offsetting, but their use as carbon credits to compensate for fossil fuel emissions will need to phase out. First, in the net zero world we are moving toward, there will be no scope for large-scale compensation for emissions through the purchase of avoided emission and emission reduction carbon credits, because those reductions would have already occurred on the journey to net zero, and because, by definition, a net zero state is one in which remaining emissions are balanced with exclusively *removals*. All *avoided emission* carbon credits, including both nature-based avoidance and fossil fuel displacement, will therefore need to be phased out as a means of compensating for ongoing fossil fuel emissions, even as the activities that underpin them scale up. Some limited use of emission avoidance credit-like instruments may persist after global net zero is achieved, perhaps as a means for facilitating abatement financing by countries with net negative emissions toward countries that still have net positive emissions, but they will by definition not be useable for making compensation claims. While nature-based *removal* carbon credits will continue to play an important role throughout the net zero transition, they face several constraints including limited land area in competition for food and fibre production, and the impact of global warming itself which is likely to substantially weaken, if not reverse, many biospheric carbon sinks (Brienen et al. 2015; Lowe and Bernie 2018). Again, within the context of those risks, these underlying activities will need to be maximally scaled in order to preserve ecosystem integrity, to preserve the ongoing capacity of nature to absorb carbon, and to deliver critical non-carbon co-benefits (Girardin et al. 2021). However, the narrow and specific use case of packaging these activities into carbon credits that claim to compensate for *fossil fuel* emissions is incompatible with a durable net zero state (Fankhauser et al. 2022; Worthington et al. 2021). Using these activities to make *contribution* claims, or to make compensation claims for biogenic emissions, is not at issue nor considered in this paper.

For continued compensation for fossil fuel emissions to be compatible with a transition to durable net zero emissions, an increasing fraction of the carbon that underpins those carbon credits must be allocated to carbon storage options that are likely to persist on long-term, geological timescales with negligible risk of reversal to the atmosphere (Allen, et al. 2020). Initially that permanently stored carbon could come from a mix of emission reductions (e.g. equipping carbon capture and storage to existing emission sources) and carbon removal methods (e.g. mineralisation, direct air capture with carbon storage), but as the net zero date approaches, a full transition to exclusively removals will be necessary. See the Oxford Principles for Net Zero Aligned Offsetting (Allen et al. 2020) for a discussion of the dual transitions from emission reduction to removal, and from low to high durability carbon storage, and for a taxonomy of carbon credit types which we refer to in this article. We propose that this transition can be packaged into a new offsetting instrument, termed a progressive offset or “proset”, generating demand for effectively permanent CO_2_ storage at an affordable cost while not undermining the strong case for immediate investment in shorter-term storage, much of which relies on critically needed nature-based climate solutions.

Offsetting is the act of paying a third party to compensate for the impact of one’s own emissions through one of two actions: emission reduction/avoidance or carbon removal. Emission reduction or avoided emission carbon credits are generated when a third party emits less CO_2_ relative to a counterfactual baseline (what they would have emitted in the absence of the revenues from carbon credit generation). Carbon removal credits are generated when CO_2_ is extracted directly from the atmosphere or upper ocean and stored durably (Shukla et al. 2022). For all instances of carbon removal, and for many instances of emission reduction and avoidance carbon credits, the reduced, avoided, or removed CO_2_ must be stored and maintained in a carbon stock. For example, the storage site could be in wild or managed ecosystems (forests, soils, etc.), in mineral forms or subsurface formations (carbonates, saline aquifers, disused oil and gas wells, etc.), in the oceans, or in long-lived products or the built environment.

There are many challenges with ensuring the atmospheric integrity of carbon credits—that is, whether they actually deliver a climate benefit to the atmosphere—which are documented in offsetting guides (Broekhoff et al. 2019), case studies (Cavanagh and Benjaminsen 2014), and systematic reviews (Galik and Jackson 2009; Haya et al. 2020; Cames et al. 2016). Key concerns affecting integrity include quantification (How much CO_2_ is actually avoided or removed?), constraining the risk of non-additionality (Might mitigation have taken place in the absence of demand for the credits generated by the carbon project?), indirect carbon leakage (Has deforestation in one location simply been displaced to another?), and the risk of physical reversal, sometimes referred to as durability or permanence (Will a forest remain intact in perpetuity in the face of pests, fire, logging, agricultural development, and global warming itself? Will CO_2_ stored in the subsurface escape before it is chemically immobilised? Does the carbon credit delay rather than permanently avoid emissions? What is the risk that stored CO_2_ will be re-emitted to the atmosphere, and if so, how soon?). All of these criteria must be rigorously enforced to ensure integrity of the purported carbon benefit, and any carbon credit used for the purpose of transitioning to and ultimately delivering net zero emissions must represent an unequivocal, high-certainty change in atmospheric CO_2_ that has a low risk of non-additionality, is free from indirect carbon leakage, and is well accounted for.

However, for the purposes of constructing a net zero-aligned “proset”, we assume for the moment that these other criteria are met, and are chiefly concerned with the question of the *durability* of stored carbon, or the resistance of stored carbon to being re-released to the atmosphere. CO_2_ released by fossil fuel combustion represents the addition of carbon that was previously preserved safely in the lithosphere into a much more labile form, circulating freely in the atmosphere, ocean, and biosphere, and elevating global temperatures for thousands of years. Therefore to be fully effective, any carbon storage intended to compensate for fossil fuel emissions must, in effect, be equally permanent. “Physical” permanence can be delivered by employing CO_2_ storage techniques with extremely low physical risks of reversal, such as the chemical immobilisation of CO_2_ into mineralised forms either aboveground or in geological formations, or the incorporation of carbon into sediments. Alternatively, “virtual” or “contracted” permanence could also be delivered through financial or legal mechanisms (e.g. insurance, covenants, an accruing pool of funds) that insure that any physical reversal event must be remediated by “topping up” a comparable carbon sink. In this way, higher-risk carbon storage techniques could be made “virtually permanent”, provided trust is maintained in the institutions and legal instruments used to ensure liability for remediating reversals of CO_2_ to the atmosphere. There is a premium to the climate for carbon storage that has a negligible risk of physical reversal since it can continue to provide a climate benefit with limited human intervention even when the entities who financed the removal and storage have long since been absolved of liability or indeed have ceased to exist.

Beyond ensuring that the standard carbon credit quality criteria are met, there are three further overarching challenges that threaten to undermine the effectiveness of voluntary carbon offsetting as currently practiced. The first is the risk of perpetuating the use of predominately emission reduction (and avoided emission) carbon credits in lieu of supporting a progressive transition to carbon removal credits. For clarity, we are not referring to the act of reducing emissions, which remains the most important and urgent means of delivering progress toward net zero emissions for all actors. Absolute emission reductions at the firm or state level must remain the top priority in a mitigation hierarchy. Carbon credits should be used primarily as a means of compensating for residual, unabatable emissions, not as a replacement for cost effective direct reductions to CO_2_ emissions. Rather, we refer specifically here to the offsetting use case of emission reduction and avoided emission carbon credits, which make up a supermajority of the credits available today on the voluntary carbon market (Mitchell-Larson and Bushman 2021). These credits, even if perfectly administered, are not sustainable in a net zero world. Once global emissions reach net zero, there will be no scope to compensate for ongoing emissions by paying a third party to reduce their emissions. Use of avoided emission carbon offset credits must therefore be transitional, and ultimately give way to exclusive reliance on removal carbon offset credits to compensate for any residual emissions (Allen et al. 2020).

Second, it is not possible to compensate indefinitely for continued use of fossil fuels through carbon removal with Nature-Based Climate Solutions (NbCS, the management of natural or human-mediated biological systems such as forests, grasslands, wetlands, and increasingly agricultural soils to enhance carbon storage). Large-scale conversion of fossil carbon into biologically stored carbon cannot be sustained in perpetuity because the global biosphere’s capacity is limited (Griscom et al. 2017). Projections of the rate of release of carbon from the biosphere by mid-century, for example, through thawing tundra, changes to tropical forest carbon fluxes, or increased wildfires, are similar to optimistic estimates of the potential global rate of carbon uptake by NbCS (Lowe and Bernie 2018). It is therefore possible that all available NbCS options will be required simply to prevent the global biosphere from further exacerbating global warming, leaving no additional capacity to compensate for ongoing fossil fuel emissions. The use of NbCS to compensate for fossil fuel emissions specifically (as opposed to biogenic emissions) taps into a rapidly depleting global resource which is under fierce competition from other land uses, primarily agriculture and timber management to provide food and fibre (Mackey et al. 2013; Dooley and Kartha 2018).



Despite these challenges, the global market for voluntary offsets approached 100 million metric tonnes of carbon dioxide equivalent (MtCO_2_e) in 2018 at an average price of around $3 per tonne (Hamrick and Goldstein 2016), almost all of which represent avoided emissions and carbon storage in relatively high-risk carbon stocks (Mitchell-Larson and Bushman 2021). This highlights a third systemic problem with the voluntary carbon market as currently constituted: carbon credit prices are typically too low to motivate buyers to reduce their own emissions, locking in high-carbon behaviour and investment. Relatedly, 75% of carbon projects to date operated outside of North America and Europe, predominately in the Global South (Mitchell-Larson and Bushman 2021), and sold their credits to what were likely wealthier, polluting entities in OECD countries. The trading of these very inexpensive permits to pollute can raise issues of equity, disenfranchisement of local communities (Lejano et al. 2020; Finley-Brook 2016), and the exhaustion of “low-hanging fruit” abatement opportunities in countries that may have few other means of meeting climate goals.

Growing awareness of these problems with traditional offsetting has fuelled interest in specialised products that compensate for the impact of fossil fuel emissions by capturing and storing CO_2_ for very long timescales. This interest has so far focused primarily on nascent carbon removal pathways such as direct air capture of CO_2_ coupled with geological carbon storage (DACCS), remineralisation (converting CO_2_ into rock), and various forms of biomass carbon removal and storage (BiCRS (Sandalow et al. 2020)), whose maturity and theoretical potential have been reviewed elsewhere (Smith et al. 2016; Fuss et al. 2018). These are currently much more expensive than traditional carbon credits (e.g. $300–700/t CO_2_ for DACCS (Izikowitz 2021; Bui et al. 2018), $50–500 for mineralisation (Kelemen et al. 2019)). In principle, conventional carbon capture and storage at currently unabatable industrial point sources (CCS), while an emission reduction and not carbon removal, provides the same atmospheric outcome and security of storage as the above examples, and typically at significantly lower cost due to the much higher CO_2_ concentrations in industrial flue gasses as compared to the ambient atmosphere (Bui et al. 2018). Indeed, most offshore CO_2_ storage infrastructure in development will likely accommodate a mix of both removed CO_2_ and CO_2_ from these applications (emission reductions). However, conventional CCS faces deeper questions about its additionality when packaged into voluntary carbon credits, given overlapping incentives from other industrial and climate policies, as well as challenges to its public acceptability (Cox et al. 2020). We therefore expect emission reductions with permanent storage (e.g. CCS) to be less readily deployable than carbon removal with permanent storage (e.g. DACCS) into the voluntary carbon market. For this reason, we further propose below that for the time being, prosets be composed of exclusively carbon removal credits.

In summary, any entity aiming to compensate for the impact of its fossil fuel emissions in the next few years is faced with an uncomfortable choice among (1) relatively cheap, but in many cases ineffective traditional carbon credits (predominately avoided emissions); (2) moderately more expensive carbon removal credits with relatively insecure storage, or emission reduction credits with highly secure storage; and (3) carbon credits that are both removals *and* deliver highly secure storage (e.g. DACCS), but which remain expensive and scarce for now.

## Prosets: a progressive transition to higher-durability CO2 storage

The solution we propose is to define a new financial instrument, named a progressive offset or “proset”, which allows the purchaser to compensate for the impact of their emissions by physically storing an equivalent quantity of CO_2_ in a predetermined mix of carbon stocks including the biosphere (primarily vegetation and soils) and lithosphere (the earth’s crust), and potentially the ocean, long-lived products, and the built environment. The defining characteristic of a proset is that the minimum fraction of CO_2_ that is stored in a highly durable format increases progressively over time, following a path that is defined by the proset itself. High-ambition buyers may choose to include more higher-durability storage than the minimum required fraction. In this context, higher-durability storage means storage of carbon in reservoirs with a risk of physical reversal of less than 0.01% per year, predicting an effective residence time of greater than 10,000 years, achievable in subsurface storage where CO_2_ in pore spaces is physically and chemically immobilised over time (García and Torvanger 2019). Such storage is as close to “physically permanent” as possible, though ongoing monitoring and legal provisions to ensure remediation and “contractual permanence” in the event of a reversal are still required. The remaining carbon credits in a proset are made up of lower-durability stored carbon in reservoirs with an expected residence time of greater than 100 years, which would likely be predominately in well-managed forests and other managed or wild ecosystems, or perhaps as elements of the built environment, biochar, or other intermediate-duration storage pathways.

**Fig. 1:**
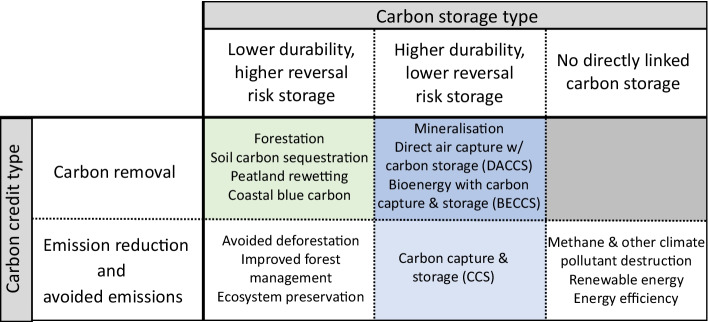
This simplified taxonomy of carbon credits distinguishes between removal and emission reduction/avoided emission carbon credits on the one hand and by the character of the carbon storage employed on the other hand. Indicative, non-exhaustive examples of carbon project types for each category are shown. Carbon project types with higher-durability, geological-timescale storage (which makes up an increasing percentage of a “proset”) are shaded in blue. Carbon removal with lower-durability storage is shaded in green. Note that “lower durability” does not mean impermanent; despite having in general higher reversal risks, these carbn storage methods can be de-risked and made “contractually permanent” through financial and legal mechanisms

**Fig. 2:**
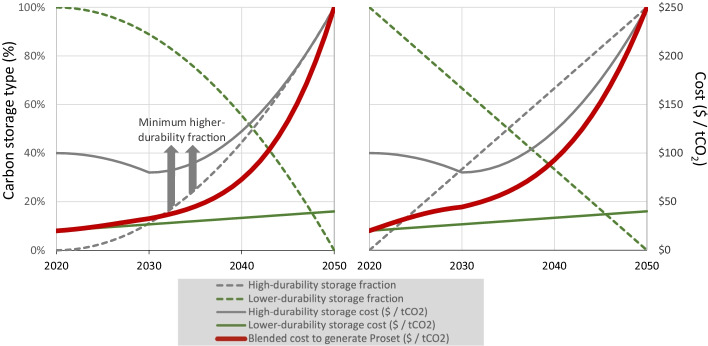
CO_2_ storage method and cost for 2020–2050s- and first-order removal prosets. The figure depicts two illustrative removal prosets, a second-order (quadratic) 2020–2050 proset (left) and a first-order (linear) 2020–2050 proset (right). See main text for full definition. Percentages of lower-durability (primarily biosphere) and higher-durability (primarily lithosphere) storage shown as green and grey dashed lines, respectively. The cost (solid lines) of lower-durability storage is assumed to escalate linearly from $20/tCO_2_ in 2020 to $40/tCO_2_ in 2050, representing an approximate cost for forestation carbon credits that increases over time as the cheapest land is exhausted. These costs are in line with recent carbon removal procurements with a high share of forest carbon storage (e.g. Microsoft’s 2021 carbon removal procurement (Joppa 2020)). Higher-durability storage costs represent the assumed full-chain cost to remove, transport, and store 1 t of CO_2_. This is assumed to start at an initial cost of $100/tCO_2_ in 2020, representing a blend of carbon removal methods involving the capture and storage of high-purity streams of biogenic CO_2_, such as biogas separation, fermentation processes, and waste treatment (Bui et al. 2018; Irlam 2017). We assume that these costs initially begin declining toward $80/tCO_2_ in 2030 due to learning-by-doing as carbon storage projects begin to scale up. From 2030 to 2050, the dominant factor in the cost evolution is assumed to be the declining availability of cheaper, higher-purity point sources of CO_2_ leading to an ever-increasing reliance on lower-purity streams of CO_2_, including direct removal from the atmosphere. We therefore assume that by 2050, the steady-state cost of a blended portfolio of carbon removal with ultra-low risk of reversal (e.g. DACCS, mineralisation) approaches a backstop cost of $250/tCO_2_ reflecting the conservative end of claims of feasible long-term DACCS costs (Bui et al. 2018; Shayegh et al. 2021; Lackner and Azarabadi 2021). Forward-looking cost assumptions are inherently uncertain, not meant to be predictive, and not inclusive of the effects of demand. However, the general trend for a proset will hold: initial cost is low and mirrors the cost of biological carbon removal, but trends upward toward a backstop cost reflecting the future cost of higher-durability carbon removal. Arrows are included to indicate that the higher-durability fraction of a proset can be higher than the minimum threshold depending on the ambition of the buyer, but it must be above that escalating minimum threshold

The time evolution of the higher-durability stored fraction is specified in the full definition of a proset, which includes the start date, the end date (which is the net zero date), and the order of the polynomial describing the increase in the higher-durability storage fraction. In a second-order 2020–2050 proset (illustrated in Fig. 1), for example, this higher-durability fraction increases with time from 2020, divided by the time from 2020 to 2050, and raised to the power of two (hence second order). The higher-durability storage fraction would therefore be (2/30)^^2^, or 0.4%, in 2022; (10/30)^^2^, or 11%, in 2030 and (20/30)^^2^, or 44%, in 2040. By 2050, the specified net zero date, it would always reach 100%. This is a general definition of a proset, but if only the end date is given, it must be assumed that the initial date is fixed to 2020, not the date on which prosets are adopted (to avoid unfairly benefiting late adopters). Illustrative examples of 2020–2050 prosets are shown in Fig. 2.

**Fig. 3:**
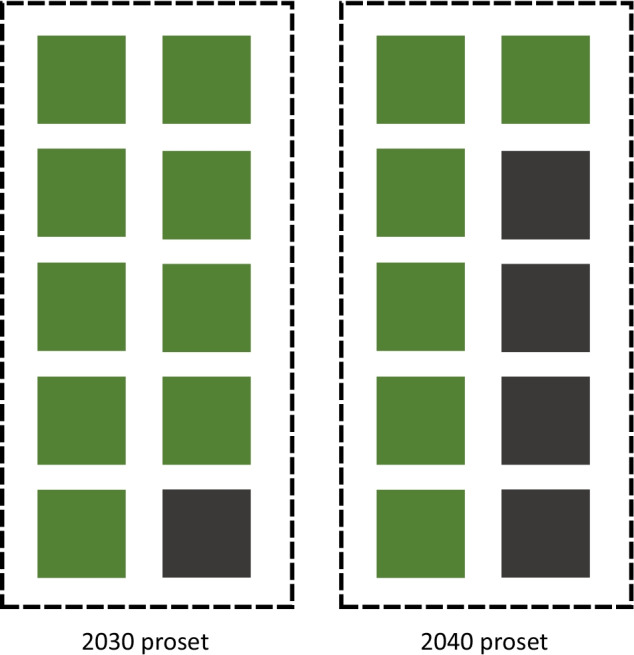
Prosets packaged for given years are in practice created by aggregating a mix of lower-durability storage (green) and higher-durability storage (grey) carbon credits. Shown here are illustrative prosets intended for retirement in the years 2030 (roughly 10% higher-durability storage) and 2040 (roughly 40% higher-durability storage) of a 2020–2050s-order proset trajectory. If this 2030 proset went unsold and unretired, its constituent carbon credits could be repackaged in another year (with the appropriately escalated higher-durability storage fraction)

A proset is a packaged product made up of a blend of constituent carbon credits with different storage attributes. The blend of credits making up a proset is determined by the year in which the proset is to be retired. As a result, prosets that are assembled, sold, and retired in 2025 could not be retired in another year, because subsequent years require a higher percentage of higher-durability storage. However, unsold 2025 prosets could be broken up into their constituent carbon credits, and repackaged into new prosets with the appropriate (and increased) percentage of higher-durability carbon storage for a subsequent year (see Fig. 3). Whereas unretired conventional carbon credits of a given “vintage” can persist from year to year until they are eventually retired, prosets must be packaged and sold within a single year. Proset providers are essentially portfolio managers responsible for purchasing requisite volumes of both lower- and higher-durability storage carbon credits to package into prosets for each year.

Note that this general definition of a proset is agnostic to the source of CO_2_ for both the higher-durability and lower-durability storage components. In practice, the entity governing the permissible composition of a proset (see discussion below) will need to precisely define which sources of CO_2_, and which storage methods are to be included. A critical decision will be whether to limit the proset to exclusively carbon removal credits. Given the challenges of developing a market for higher-durability CO_2_ storage, we see an argument for allowing any.

CO_2_ that would otherwise, under normal business practice, have ended up as unabated emissions into the atmosphere, to count towards the stored fraction of a proset. This would allow CO_2_ capture at fossil CO_2_ point sources, which is emission reduction and not removal, to be utilised, provided this was not already being used to discharge some other obligation such as compliance with an emission trading system (which would compromise additionality). As point sources are brought under progressively tightening emission caps, an increasing fraction of permanently stored CO_2_ would inevitably need to be sourced from carbon removal methods such as mineralisation, direct air capture, or remaining instances of biogenic CO_2_ capture coupled with geological storage (see the BiCRS roadmap for an overview of biogenic carbon removal including higher-durability methods (Sandalow et al. 2020)). For the lower-durability component, because the proset is composed of carbon credits representing a physical flux of CO_2_ into storage, the only carbon credits available will be carbon removal.

While we could justifiably assume that the transition in the higher-durability storage fraction from primarily point source emission reduction toward primarily carbon removal would occur organically, proset adopters might choose to further specify the breakdown in the source types of CO_2_ used to generate their prosets. For example, some users might require that a progressive fraction of the higher-durability storage component used to create their proset come from carbon removals rather than emission reductions. This would have the effect of providing targeted support for remineralisation methods, enhanced weathering, various forms of high-durability BiCRS, and DACCS—all carbon removal methods which will benefit from early investment to achieve scale.

From a climate outcome standpoint, emphasis is most usefully placed on the character and security of carbon storage (higher vs. lower durability), not the source of the CO_2_ (removals vs. reductions). However, keeping proset composition limited to exclusively carbon removal credits makes net zero claims possible, as avoided emission and emission reduction credits cannot be used to claim to have achieved net zero (Allen et al. 2020). A removal-only proset also sidesteps some additionality questions given overlapping policy incentives for industrial abatement, and some of the issues of public acceptability faced by conventional carbon capture mentioned above. In Fig. 2, we therefore provide an illustrative example of a removal-only proset. The proset model can easily accommodate additional exclusion criteria provided the essence of the concept—a fundamental progression toward 100% higher-durability and physically permanent storage—is maintained.

By definition, a commitment to purchase prosets to cover ongoing fossil fuel emissions provides a predictable pathway to durable net zero by the target date, while also compensating for the warming impact of those emissions in the meantime, consistent with both the letter of corporate commitments to achieve net zero emissions as soon as possible, and also with the spirit of these commitments to sustainably end their contributions to global warming. It does so at a cost that we estimate is, in the early 2020s, no higher than that of many conventional, high-quality carbon credits, but which will increase as the net zero date approaches (see Fig. 2).

In principle, as long as the higher-durability storage fraction minimum rises to 100% or greater by the proset end date, this provides a transition path to net zero regardless of the shape of the evolution of this stored fraction. But the higher the order of the proset, the more the effort is backloaded. A third-order 2050 proset would require only (1/3)^3^, or 3.7%, permanent storage in 2030. An observed result of least-cost ambitious mitigation scenarios from integrated assessment models (Huppmann et al. 2019) is that the fraction of global CO_2_ production that is permanently stored increases approximately quadratically, as in a second-order proset, from 2020 to the date of net zero, though we would call for renewed analysis using updated cost and availability assumptions now that the likely availability of various carbon removal methods is better understood (Jenkins et al. 2021). Further backloading this increase through the use of third or higher-order prosets would therefore impose a disproportionate fraction of the cost onto future decades, undermining the credibility of the commitment.

This transition to durable net zero will need to be codified in policy, for example, in compliance carbon markets, with mandates, or through other regulatory instruments. Commitments to buy prosets are initially voluntary, which is a suboptimal level of assurance that participants will follow through in later years as the makeup of the prosets change and their cost increases.

## The case for universally defined prosets

Although it has been used to fund some laudable initiatives, the voluntary carbon market has thus far failed to deliver a net zero compliant instrument. Supply has greatly exceeded demand, and many standards have been overly lax, resulting in very low prices and carbon credits of dubious quality. There are many competing standards, none of which addresses the need to transition to higher-durability storage for compensating fossil fuel emissions specifically. Many carbon credit sellers already allow purchasers to specify a mix of carbon credit types, but that mix is determined by purchasers’ preferences, not by what a durable net zero pathway requires.

The fact that the fraction of CO_2_ produced by the burning of fossil fuels globally that is permanently stored needs to rise to 100% by the time of global net zero emissions has long been noted (Allen et al. 2009), but the concept of a time-evolving permanent storage fraction built into the design of an offset-like product, with a pre-specified profile to achieve durable net zero by a specific date is, to our knowledge, novel.

While recognising the dangers of a proliferation of terms, we believe that introducing a new word to define this concept may be helpful to avoid the definition “race-to-the-bottom” that often besets conventional carbon credits and offsetting. The definition we propose is sufficiently general that it would apply to any monotonically increasing higher-durability storage fraction, and hence to any offsetting programme that is genuinely compatible with a transition to durable net zero. While we note the dangers of excessive backloading, we prefer to avoid freezing the definition of the rate of escalation of the higher-durability storage fraction from the outset, hoping to contribute to the ongoing discussion about the role of offsetting in the net zero transition.

A possible way forward would be for a coalition of scientists, environmental NGOs, and carbon market participants to work together toward a universally acceptable, net zero compliant proset definition, including both the profile (or order) of the proset and the permissible characteristics of both the CO_2_ storage options and the emissions that the proset can be used to compensate for. Such an initiative could be stewarded by an established organisation or initiative. The advantage of a centrally defined proset is that it could potentially be used to prevent the sale of lower-quality, proset-like products that would undercut the market by adopting a looser definition of “higher-durability storage”, or a late-increasing profile that backloads the transition to higher-durability storage. The disadvantage is that it might discourage some adopters, particularly if prosets were strongly associated with a single profit-making supplier or certifying agency. This could be addressed by making a clear distinction between the supply and assembly of prosets, and the certification of prosets, and entrusting a not-for-profit, charitable entity with a binding mandate to maintain proset integrity.

In terms of the broader role of carbon offsetting in the fight against climate change, we agree with Stephen Schneider’s assessment: “I don’t believe offsets are a distraction. But we’ll have failed if that’s all we do.” (Ellison 2007) Volunteerism has its limits, and concerted climate action eventually necessitates regulation. But voluntary carbon markets are more than just a placeholder. They can provide a testing ground for compliance markets, leading to regulation which uses voluntary action as evidence for what is possible. Prosets specifically offer a means of transitioning to higher-durability storage on a voluntary basis, a transition which we envision will be taken up by policymakers who wish to codify and enforce a balance of extracted carbon with permanent sinks (Allen et al. 2009), to deliver durable net zero.
